# Estimating the incidence of lung cancer attributable to occupational exposure in Iran

**DOI:** 10.1186/1478-7954-7-7

**Published:** 2009-05-12

**Authors:** Alireza Mosavi-Jarrahi, Mohammadali Mohagheghi, Bita Kalaghchi, Yasaman Mousavi-Jarrahi, Mohammad Kazem Noori

**Affiliations:** 1 Dept of Public Health and Social Medicine, Shaheed Beheshti University of Medical Sciences and Health Services, Tehran, Iran; 2 The Cancer Institute Research Center, Tehran University, Tehran, Iran; 3 Dept of Health Economic, Medical School, Shahed University, Tehran, Iran; 4 Environmental health and safety office, The Bureau of Development and Renovation organization of Mine and Mineral industries, Tehran, Iran

## Abstract

**Objective:**

The aim of this study was to estimate the fraction of lung cancer incidence in Iran attributed to occupational exposures to the well-established lung cancer carcinogens, including silica, cadmium, nickel, arsenic, chromium, diesel fumes, beryllium, and asbestos.

**Methods:**

Nationwide exposure to each of the mentioned carcinogens was estimated using workforce data from the Iranian population census of 1995, available from the International Labor Organization (ILO) website. The prevalence of exposure to carcinogens in each industry was estimated using exposure data from the CAREX (CARcinogen EXposure) database, an international occupational carcinogen information system kept and maintained by the European Union. The magnitude of the relative risk of lung cancer for each carcinogen was estimated from local and international literature. Using the Levin modified population attributable risk (incidence) fraction, lung cancer incidence (as estimated by the Tehran Population-Based Cancer Registry) attributable to workplace exposure to carcinogens was estimated.

**Results:**

The total workforce in Iran according to the 1995 census identified 12,488,020 men and 677,469 women. Agriculture is the largest sector with 25% of the male and 0.27% of female workforce. After applying the CAREX exposure estimate to each sector, the proportion exposed to lung carcinogens was 0.08% for male workers and 0.02% for female workers. Estimating a relative risk of 1.9 (95% CI of 1.7–2.1) for high exposure and 1.3 (95% CI 1.2–1.4) for low exposure, and employing the Levin modified formula, the fraction of lung cancer attributed to carcinogens in the workplace was 1.5% (95% CI of 1.2–1.9) for females and 12% (95% CI of 10–15) for males. These fractions correspond to an estimated incidence of 1.3 and 0.08 cases of lung cancer per 100,000 population for males and females, respectively.

**Conclusion:**

The incidence of lung cancer due to occupational exposure is low in Iran and, as in other countries, more lung cancer is due to occupational exposure among males than females.

## Introduction

With the rise in the incidence of occupational lung cancer, information is increasingly available for developing countries to use as a tool to combat the preventable causes of this disease. One source of such information is the CAREX (CARcinogen EXposure) international database on occupational exposure to known and suspected carcinogens. Constructed with the support of the program Europe Against Cancer of the European Union, CAREX provides selected exposure data and documented estimates of the number of exposed workers organized according to carcinogen and industry. Another source, the International Agency for Research on Cancer (IARC)[1], has classified 87 agents as carcinogenic to humans (Group 1), the majority of which have been associated with lung cancer. Among the many carcinogens in IARC Groups 1 and 2A, silica, cadmium, nickel, arsenic, chromium, diesel fumes, beryllium and asbestos are established lung carcinogens. Epidemiologic studies of these carcinogens have reported high and moderate magnitudes of relative risk for lung cancer among workers exposed to each of these workplace carcinogens.

Lung cancer is mainly caused by smoking, in addition to other environmental carcinogens, especially those in the workplace. Determining what fraction of lung cancers are due to workplace exposure to known carcinogens has been a challenging issue since late 1970, when scientists of the National Cancer Institute, the National Institute of Environmental Health Sciences, and the National Institute for Occupational Safety and Health of the United States estimated that up to 20%–40% of all cancers were the result of occupational exposures[2]. In 1981, Doll and Peto estimated that the fraction of cancer in the United States attributable to occupational exposure ranged between 2% and 8%[3], a very sharp difference from that of the previous estimate. Since then, several authors have estimated the impact of exposure to carcinogens in the workplace on the total burden of cancer in several countries, using different methodologies to estimate the burden of cancer incidence due to occupational exposure. Among these methodologies, the attributable risk concept (the fraction of disease occurring in a given population that would not have occurred had the factor of interest been absent), introduced by Levin[4] in 1953, has been of prime interest to epidemiologists and public health scientists. Attributable risk can be measured either among the exposed or in the general population (population attributable risk or PAR) [5-7]. The PAR fraction depends on the magnitude of the association between an exposure and a disease, as well as on the prevalence of the exposure in the population. The incidence of lung cancer in Iran is reported to be 12 per 100,000 population[8]. The smoking prevalence in Iran is relatively low, with 24% of the population having ever smoked[9], and a sizable fraction of lung cancer incidence may be the result of environmental and workplace exposure to lung carcinogens other than smoking.

Using published figures regarding the relative risk of lung cancer associated with exposure to the major workplace lung carcinogens (silica, cadmium, nickel, arsenic, chromium, diesel fumes, beryllium, and asbestos) and the proportion of exposed workers in different workforce sectors, this study aimed to estimate the fraction of lung cancer incidence in Iran attributable to occupational cancer. This study is the first published estimate of a nationwide incidence of occupational lung cancer in the Middle East.

## Methods

The methodology involves four main steps: 1) estimating the prevalence of exposure at any time in the workforce for the carcinogens of interest, including silica, cadmium, nickel, arsenic, chromium, diesel fumes, beryllium and asbestos; 2) estimating the magnitude of relative risk of lung cancer associated with the carcinogens of interest; 3) estimating the attributable fraction using Levin's formula; 4) calculating the fraction of lung cancer incidence attributed to occupational exposure from the reported incidence in population-based cancer registries.

To estimate the prevalence of exposure in the Iranian population to the eight above-mentioned carcinogens, the number of workers (both male and female, over 15 years of age) employed in each economic sector was obtained from the International Labor Organization (ILO) online workforce databases[10]. Non-overlapping exposures were assumed among the carcinogens of interest. It was assumed that the exposures were independent and their joint effects were additive. The association of multiple exposures with the same cancer site and the methodology to handle such exposure scenarios in estimating attributable fraction has been extensively addressed in epidemiologic literature [11-14]. At two recent international workshops ;  held to tackle the methodology pertaining to occupational carcinogens assessment, a non-overlapping exposure scenario (when multiple exposures cause the same cancer) was recommended and in the case of an overlap, it was recommended to estimate the attributable fraction just for the dominant exposure or the overlapping exposures be treated as separate exposure scenarios (an exposure set) handling the interaction within the exposure set. The CAREX estimated proportion of exposed workers for each industry and carcinogen[15,16] was applied to the number of male and female workers in each industry. Since the number of workers in each industry was based on the 1995 national census, the proportion of workers who had ever been exposed was obtained by multiplying the proportion of those currently exposed by the occupational turnover rate (the annual replacement of workers in a given job) as well as the rate of the economically active population. Due to lack of an official worker turnover rate for Iran, the recommended average worker turnover of 4 was used[17]. The rate of the economically active population for Iran (74.4% for males and 28.6% for females) was obtained from the ILO web site[10].

The reported relative risk of lung cancer for exposure to the carcinogens of interest is estimated to be 1.3 (95% CI: 1.2–1.4) for low-level exposures, and 1.9 (95% CI 1.7–2.1) for high-level exposures to lung carcinogens[18]. As recommended, a partition factor of 0.5 was used to divide the number of exposed workers into low and high levels of exposure[17], since in developing countries a higher proportion of the workforce receives a higher level of exposure due to compromise in workplace safety standards (the recommended partition factor is 0.9 and 0.1 for low and high exposure, respectively, in developed countries). The attributable fraction or attributable incidence fraction (AIF) was calculated using the modified Levin equation (see Additional file 1).

There is not a national population-based cancer registry covering total population of the country. Instead, the Tehran Population-Based Cancer Registry (TPCR) is a large registry covering 10% of the Iranian population. The population covered by TPCR represents the total population of the country in terms of cancer rates. To estimate the fraction number of lung cancer incidence attributed to occupational exposure, the incidence of lung cancer, as reported by TPCR[8], was multiplied by the estimated AIF. The 95% confidence interval was constructed using the lower and upper confidence limits estimated for each of the relative risk estimates.

## Results

A total of 12,488,020 males and 677,469 females were employed in different industrial sectors of Iran in 1995. Agriculture was the largest sector for males (25%) and the service sector was the largest one for females (90%), with high employment rate among different industrial sectors. Table 1 presents the number and proportion of males and females in each industrial sector based on the 1995 census. The CAREX estimate of the proportion of workers exposed to the eight major workplace lung carcinogens in each industry is presented in Additional file 2. Applying the matrix of the carcinogen and exposure proportion to the number of workers in each industry provided the proportion of the workforce exposed to each carcinogen for each sex (see Additional file 2). In total, 8% and 2% of the male and female workforce, respectively, were exposed to the carcinogens of interest. Application of the national turnover and the growth rate of the economically active population to the proportion of exposed, considering a 50% partition factor for low and high level exposure, shows that 12% of males and 1% of females receive low-level exposure and the same proportion receives high-level exposure. Employing the prevalence of those who have ever received low- and high-levels of exposure and their corresponding risk ratios, the Levin formula produces estimates of 12% among males and 1.5% among females for lung cancer incidence attributed to the carcinogens of interest. However, applying the attributable fraction to the actual estimate of lung cancer reveals a work-related incidence of lung cancer of 1.3 (95% CI 1.1–1.6) per 100,000 population for males and 0.08 (95% CI of 0.06–0.09) per 100,000 population for females, as shown in Table 2.

**Table 1:** Number and proportion of Iranian workers in each economic sector according to sex*

	Male		Female	
	
Sector	No.	Percent	No.	Percent
Agriculture	3062798	24.53	1827	0.27
Mining	115185	0.92	4014	0.59
Manufacturing	1968806	15.77	24000	3.54
Electrical	145239	1.16	4475	0.66
Construction	1634682	13.09	1413	0.21
Trade	1804143	14.45	4143	0.61
Transportation	955271	7.65	9258	1.37
Finance	139286	1.12	12111	1.79
Services	2662610	21.32	616228	90.96

Total	12488020	100	677469	100

*based on the 1995 census, ILO 2006

**Table 2:** Estimated attributable fraction and corresponding incidences

Sex	lung cancer incidence^a^	Estimated attributable fraction for occupational exposure (95% confidence interval)	Estimated incidence (95% confidence interval)
Male	10.4	0.12 (0.10, 0.15)	1.30 (1.10, 1.60)
Female	5.01	0.014 (0.011, 0.018)	0.08 (0.06, 0.09)

^a^Based on data from the Tehran Population-Based Cancer Registry.

## Discussion

Our study estimated the portion of lung cancer incidence in Iranian workers attributed to major lung carcinogens. The carcinogenicity and the magnitude of association between the carcinogens and lung cancer have been subject of several reviews. A recent systematic review of epidemiological investigations on silica exposure and lung cancer risk, including 28 cohort studies, has estimated a pooled relative risk of 1.69 among exposed workers[19]. Arsenic is another well-studied lung carcinogen with a reported relative risk of 3.69 (95% CI of 3.06 to 4.46)[20,21]. Beryllium is another occupational lung carcinogen with a relative risk of 1.24 among workers with low exposure and an SMR of 2 among a registered cohort of women[22]. Cadmium, found in cadmium-smelters, battery production, cadmium-copper alloys, dyes and pigment production, and electroplating processing plants, has an estimated relative risk of 1.2 to 1.49 for lung cancer among exposed workers[20,23]. Asbestos, a lung carcinogen and sole cause of mesothelioma found mainly in the mining and milling, by-product manufacturing, insulation and construction industries has a relative risk of at least 2.0 for lung cancer among those with moderate to high level exposure[20,23]. Categorized as a Group 2A carcinogen, diesel exposure is especially severe among truckers, railroad workers, professional drivers, dock workers[20,23-25], and mechanics, with a relative risk of 1.3 to 1.5 for lung cancer among exposed workers. Nickel, another IARC Group 1 carcinogen found in nickel mining, metal fabrication, grinding, electroplating, and welding industries, has an estimated relative risk of 1.56[20,23].

Our study estimates that 12% of the lung cancer incidence among males and 1% among females is attributed to exposure of the Iranian population to the major occupational lung carcinogens (as defined by the IARC). Although there are no published estimates for the incidence of occupational lung cancer in Middle Eastern countries (ours is the first reported attempt), the World Health Organization (WHO) global burden of disease series has estimated an attributable fraction of 11% for males and 3% for females for countries in the regions of western Asia and northern Africa[17,26].

There have been many local and international efforts in different countries to estimate the proportion of lung cancers attributable to occupational lung cancer, resulting in estimates from 3% to 26% in South Australia[27], 29% in Finland[28], and another estimate of 29% for men and 5% for women in Finland[23]. In our study, the estimated attributable fraction for males is more consistent with the published estimates from other countries than the estimate for females, as most of the estimates for females are more than 3%. A low attributable fraction among women in our population is probably due to the low prevalence of exposure and/or the small number of economically active women (as is the case in conservative countries of the Middle East), resulting in a low proportion of exposure, hence a low attributable fraction.

Although there is some heterogeneity across studies in the exposure assessments and classifications, a sizeable fraction of the variations seen in the above estimates might be due to uncertainties incorporated into estimates resulting from the methodology used and the nature of the exposure to carcinogens in different populations. The methodology used in our study has been widely used in different populations. It basically relies on the principles of estimating the attributable fraction as a function of exposure prevalence and the magnitude of the association between exposure and disease. The prevalence of exposure in our study was estimated based on data from the CAREX database, which includes data on 139 agents evaluated by the IARC (all agents in Groups 1 and 2A, and selected agents in Group 2B), displayed across the 55 industrial classes of the International Standards of Industrial Classification system revision 3. Specific to occupational exposure among Finnish and US workers, which may differ from exposure in other countries, the CAREX database has been used by several epidemiologists and the WHO to estimate the global burden of occupational cancer, and has been widely used as a basis to estimate the exposure prevalence for different countries after adjusting for certain population specificities using expert opinions and reviews[17,26,29,30].

In this study, the use of the CAREX exposure matrix may introduce some degree of uncertainty resulting from the differences in the industrial substructure of Iran compared to those of Finland and the US, such as the issue of asbestos for construction materials banned in early 1980 in Iran and smoking in the workplace, which is not yet regulated in our population. Another uncertainty in our estimation originates from the fact that the level of exposure was arbitrarily chosen as above 50% being high exposure and below 50% being low exposure. Determining what percent of the workforce receives high-level exposure depends on the regulations, standards, and the safety and exposure monitoring practices as well as the technology used to minimize the exposure or monitor its quantity and quality. As a developing country, Iran is in the midst of transformation from a primarily agricultural society to a more industrial society, and may experience varying degrees of ambiguity in its workforce regulation and industrial exposure management policy. Another uncertainty built into this kind of analysis is the classification used in the study. Our data were obtained from the ILO web site where conversion of the local workforce data into the International Standards of Industrial Classification is performed by expert review rather than at the data collection point, due to the different labor classifications used in various countries. International classifications of local data have been extensively used in epidemiological studies and its advantages and disadvantages have been well addressed[31].

Another parameter that plays an important role in our estimate is the relative risk used for the association of lung cancer with carcinogens of interest. The relative risk estimates used in our study are based on a mean relative risk of 1.6 partitioned into a relative risk of 1.3 for low-level exposure to lung carcinogens and 1.9 for high-level exposure. This has been done by calculating a mean average of the carcinogen-specific relative risks weighted by the proportion of workers exposed to each carcinogen[18,26]. As the magnitude of association between an exposure and outcome theoretically depends on the dose-response relationship between the exposure and the outcome, the source of any uncertainty would be the partitioning factor used to divide the exposed into these low and high levels of exposure.

The use of lung cancer rates from the Tehran population based cancer registry as estimates for the whole country is based on the fact that the Tehran population consists of immigrants from all over the country (all ethnic groups) who have immigrated and settled in the Tehran metropolis during the last 50 to 60 years. They form a fairly representative population of the whole country [32].

Actually measuring the incidence of disease in the workplace requires systematic disease and exposure registries as well as many resources, strong administrative policies and political will. Therefore, the methodology used in our study is well justified and considered to be one of the best approaches to estimate the burden of occupational cancer, including lung cancer in countries in which resources for the establishment of disease or exposure registries are scarce.

## Conclusion

Our study estimated a moderate work-related lung cancer incidence among males and a very low incidence among females in Iran. Further studies are needed to validate our estimate utilizing locally obtained exposure data.

## Competing interests

The authors declare that they have no competing interests.

## Authors' contributions

MM participated in the study's design and coordination and helped to draft the manuscript. BK conceived of the study. YMJ participated in the design of the study and performed the statistical analysis. MKN conceived of the study, participated in its design and coordination, and helped to draft the manuscript. All authors read and approved the final manuscript.

## Supplementary Material

Additional file 1 **Exhibition 1.** The Levin formula for calculation of the attributable fraction. The formula and description of its parameters. The formula was used to calculate the attributable fraction. Click here for file

Additional file 2 **Tables describing the estimated proportion exposed worker to expose to lung carcinogens according to CAREX as well as the percentage of Iranian female and male worker exposed to each lung carcinogens**. The formula and description of its parameters. The formula was used to calculate the attributable fraction. Click here for file
