# Supplementary figures and images for: Estimating the incidence of lung cancer attributable to occupational exposure in Iran
Source: Popul Health Metr. 2009 May 12;7:7. doi: 10.1186/1478-7954-7-7 (PMC2689160; doi:10.1186/1478-7954-7-7)

| Exhibition 1. The Levin formula for calculation of the attributable fraction |
| --- |
| 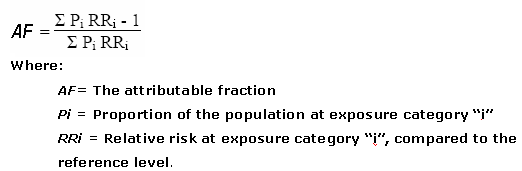 |

Supplement: Additional file 1 — Exhibition 1. The Levin formula for calculation of the attributable fraction. The formula and description of its parameters. The formula was used to calculate the attributable fraction. [file 1478-7954-7-7-S1.doc]
