# Supplementary material for: Estimating the incidence of lung cancer attributable to occupational exposure in Iran
Source: Popul Health Metr. 2009 May 12;7:7. doi: 10.1186/1478-7954-7-7 (PMC2689160; doi:10.1186/1478-7954-7-7)
Supplement: Additional file 2 — Tables describing the estimated proportion exposed worker to expose to lung carcinogens according to CAREX as well as the percentage of Iranian female and male worker exposed to each lung carcinogens. The formula and description of its parameters. The formula was used to calculate the attributable fraction. [file 1478-7954-7-7-S2.doc]

| Percentage of workers occupationally exposed to lung carcinogens according to industry.* | | | | | | | | | |
| --- | --- | --- | --- | --- | --- | --- | --- | --- | --- |
| Carcinogens | Agriculture | Mining | Manufacturing | Electrical | Construction | Trade | Transportation | Finance | Services |
| Silica | 0.004 | 0.230 | 0.023 | 0.014 | 0.189 | 0.000 | 0.005 | 0.000 | 0.001 |
| Cadmium | 0.000 | 0.000 | 0.005 | 0.003 | 0.003 | 0.000 | 0.001 | 0.000 | 0.000 |
| Nickel | 0.000 | 0.020 | 0.017 | 0.004 | 0.000 | 0.000 | 0.000 | 0.000 | 0.000 |
| Arsenic | 0.001 | 0.001 | 0.004 | 0.001 | 0.001 | 0.000 | 0.000 | 0.000 | 0.000 |
| Chromium | 0.000 | 0.003 | 0.021 | 0.004 | 0.002 | 0.000 | 0.004 | 0.000 | 0.002 |
| Diesel Fumes | 0.006 | 0.220 | 0.011 | 0.034 | 0.058 | 0.005 | 0.134 | 0.000 | 0.009 |
| Beryllium | 0.000 | 0.001 | 0.002 | 0.001 | 0.000 | 0.000 | 0.000 | 0.000 | 0.000 |
| Asbestos | 0.012 | 0.102 | 0.006 | 0.017 | 0.052 | 0.003 | 0.007 | 0.000 | 0.003 |

*Based on the CAREX database estimates, FIOSH, 1998.

| Percentage of female Iranian workers occupationally exposed to lung carcinogens according to economic sector. | | | | | | | | | |  |
| --- | --- | --- | --- | --- | --- | --- | --- | --- | --- | --- |
| Carcinogens | Agriculture | Mining | Manufacturing | Electrical | Construction | Trade | Transportation | Finance | Services | Total |
| Silica | 0.00001 | 0.00136 | 0.00081 | 0.00009 | 0.00039 | 0.00000 | 0.00007 | 0.00000 | 0.00091 | 0.00365 |
| Cadmium | 0.00000 | 0.00000 | 0.00018 | 0.00002 | 0.00001 | 0.00000 | 0.00001 | 0.00000 | 0.00000 | 0.00021 |
| Nickel | 0.00000 | 0.00012 | 0.00060 | 0.00003 | 0.00000 | 0.00000 | 0.00000 | 0.00000 | 0.00000 | 0.00075 |
| Arsenic | 0.00000 | 0.00001 | 0.00014 | 0.00001 | 0.00000 | 0.00000 | 0.00000 | 0.00000 | 0.00000 | 0.00016 |
| Chromium | 0.00000 | 0.00002 | 0.00074 | 0.00003 | 0.00000 | 0.00000 | 0.00005 | 0.00000 | 0.00182 | 0.00266 |
| Diesel Fumes | 0.00002 | 0.00130 | 0.00039 | 0.00022 | 0.00012 | 0.00003 | 0.00184 | 0.00000 | 0.00819 | 0.01211 |
| Beryllium | 0.00000 | 0.00001 | 0.00007 | 0.00001 | 0.00000 | 0.00000 | 0.00000 | 0.00000 | 0.00000 | 0.00008 |
| Asbestos | 0.00003 | 0.00060 | 0.00021 | 0.00011 | 0.00011 | 0.00002 | 0.00009 | 0.00000 | 0.00273 | 0.00391 |

| Percentage of male Iranian workers occupationally exposed to lung carcinogens according to economic sector. | | | | | | | | | |  |
| --- | --- | --- | --- | --- | --- | --- | --- | --- | --- | --- |
| Carcinogens | Agriculture | Mining | Manufacturing | Electrical | Construction | Trade | Transportation | Finance | Services | Total |
| Silica | 0.00098 | 0.00212 | 0.00363 | 0.00016 | 0.02474 | 0.00000 | 0.00036 | 0.00000 | 0.00021 | 0.03221 |
| Cadmium | 0.00000 | 0.00000 | 0.00079 | 0.00003 | 0.00039 | 0.00000 | 0.00005 | 0.00000 | 0.00000 | 0.00127 |
| Nickel | 0.00000 | 0.00018 | 0.00268 | 0.00005 | 0.00000 | 0.00000 | 0.00000 | 0.00000 | 0.00000 | 0.00291 |
| Arsenic | 0.00025 | 0.00001 | 0.00063 | 0.00001 | 0.00013 | 0.00000 | 0.00000 | 0.00000 | 0.00000 | 0.00103 |
| Chromium | 0.00000 | 0.00003 | 0.00331 | 0.00005 | 0.00026 | 0.00000 | 0.00028 | 0.00000 | 0.00043 | 0.00436 |
| Diesel Fumes | 0.00147 | 0.00203 | 0.00173 | 0.00040 | 0.00759 | 0.00072 | 0.01028 | 0.00000 | 0.00192 | 0.02614 |
| Beryllium | 0.00000 | 0.00001 | 0.00032 | 0.00001 | 0.00000 | 0.00000 | 0.00001 | 0.00000 | 0.00000 | 0.00034 |
| Asbestos | 0.00294 | 0.00094 | 0.00095 | 0.00020 | 0.00681 | 0.00043 | 0.00052 | 0.00000 | 0.00064 | 0.01343 |
